# Factors influencing the sensory profile in patients with autism spectrum disorder from 16 months to 14 years: results of an observational study

**DOI:** 10.3389/fpsyt.2026.1771832

**Published:** 2026-05-25

**Authors:** Nadia Marani, Davide di Gennaro, Raffaele Garotti, Valeria Maffettone, Rosamaria Siracusano, Maria Pia Riccio, Simone Pisano, Carmela Bravaccio

**Affiliations:** 1 Department of Maternal and Child Health, Child and Adolescent Neuropsychiatry, AOU “Federico II”, Naples, Italy; 2 Residency Program of Child and Adolescent Neuropsichiatry, Department of Medical and Translational Science, Federico II University, Naples, Italy; 3 Department of Mental and Physical Health and Preventive Medicine, University of Campania “Luigi Vanvitelli”, Naples, Italy; 4 Department of Psychology, University of Campania “Luigi Vanvitelli”, Naples, Italy; 5 Department of Medical and Translational Sciences, Child Neuropsychiatry, Federico II University, Naples, Italy

**Keywords:** adaptive function, age, autism spectrum disorder, cognitive function, sensory profile

## Abstract

**Introduction:**

Sensory processing abnormalities represent a high-impact clinical feature in individuals with Autism Spectrum Disorder (ASD). Sensory alterations can be classified by modality and behavioral responses to stimuli. The literature presents conflicting results regarding the association between the sensory profile and other clinical elements. Therefore, this study aimed to characterize the clinical variables that influence sensory anomalies in subjects with ASD.

**Methods:**

This single-center retrospective observational study selected 159 subjects with a confirmed ASD diagnosis, aged 16 months to 14 years. Data collected included clinical assessments (developmental profile, intelligence quotient, adaptive profile and autistic symptom severity) and sensory evaluations using the Sensory Profile-2. A Bayesian cumulative logit model was implemented to assess the impact of clinical variables on sensory profiles.

**Results:**

The analysis identifies adaptive functioning as the most consistent predictor of sensory outcomes, where higher functioning correlates with a reduction in atypical patterns, particularly sensory sensitivity. Conversely, autism severity and sex generally showed no significant impact, though higher cognitive functioning was specifically linked to increased sensory avoiding behaviors. Furthermore, the Bayesian analysis revealed a modifying effect of age on the sensory profile, characterized by a non-monotonic trend clustered around two distinct age peaks. The preschool period is characterized by a predominance of sensory seeking behaviors and auditory processing challenges, whereas the 8-to-12-year developmental window marks a significant shift toward hypersensitivity and avoidant patterns, particularly within the visual and oral modalities.

**Discussion:**

Overall, the analysis revealed that the adaptive profile has a significant impact on the sensory profile, consistent with existing literature. Age emerged as a key modifying factor with a non-monotonic trajectory, contrasting with previous studies that typically highlight either improvement or stability over time. Study main limitations include its retrospective and non-longitudinal nature. Therefore, further prospective longitudinal studies are necessary to explore these dynamic associations between the sensory profile and other clinical variables over time.

## 1 Introduction

Autism Spectrum Disorder (ASD) is a neurodevelopmental disorder characterized by deficits in socio-communicative and relational skills, associated with the presence of stereotyped and repetitive behavioral patterns (1).

Differences in sensory processing are highly heterogeneous, both in the pediatric and adult population, and are currently counted among the core symptoms of ASD (2, 3). Sensory anomalies also actively impact the functioning of children with ASD (increased anxiety levels, greater social avoidance and difficulty adapting, interference with daily learning) (4, 5). Furthermore, there is an interconnected and bidirectional relationship between attention and sensory processing in children with Autism Spectrum Disorder (ASD). In ASD, impairments in attentional control, particularly inhibitory gating, hinder the individual’s ability to filter out irrelevant environmental stimuli, such as background noise. When the attentional system fails to effectively suppress these non-salient inputs, a state of sensory overload occurs, which subsequently manifests as clinical hypersensitivity or hyperreactivity (6). The definition of anomalies related to sensory processing is based on the model proposed by Dunn W. (7), which identifies four sensory processing patterns: sensory seeking, sensory avoiding, sensory sensitivity and low registration. Individuals with ASD tend to manifest these behavioral patterns in a variable way, in relation both to their developmental level and to other aspects of the clinical profile (such as linguistic and motor skills) (8). A subject of recent scholarly interest concerns sex-based biological differences; emerging evidence suggests that males and females with Autism Spectrum Disorder (ASD) may exhibit distinct sensory profiles. Research indicates that females often exhibit a more pronounced sensory over-responsivity compared to their male counterparts, particularly within the tactile and auditory domains. Furthermore, the higher prevalence of social camouflaging in females may obscure core socio-communicative difficulties, leading to a phenotypic presentation where sensory atypicalities become a primary clinical indicator. Consequently, these distinct profiles necessitate a gender-informed approach to diagnostic screening, as traditional instruments may fail to capture the subtle interplay between sensory sensitivity and compensatory masking strategies, ultimately leading to delayed or missed diagnoses in the female population (9).The presence of abnormalities in sensory processing gains significant importance in cognitive development, influencing fundamental developmental domains such as attention, memory and language, as well as higher-order capacities like cognitive flexibility and control, social interaction, emotional regulation and personal autonomy (10). The impact of these alterations persists across the lifespan: in adults with ASD, dysfunctional sensory patterns have been associated not only with cognitive challenges but also with emotional dysregulation, which may manifest as aggressive behavior. Specifically, research indicates that sensory sensitivity and avoidance are robustly linked to reactive aggression and anger, while low registration profiles often correlate with proactive aggression. These findings underscore the significant predictive value of sensory processing on long-term psychological well-being and behavioral stability (11). Our study is situated within this theoretical framework, aiming to investigate which factors influence the sensory pattern of individuals with ASD from 16 months to 15 years of age, considering age, adaptive functioning, severity of autistic symptomatology and the presence of cognitive impairment as variables. This allows for the exploration of how different developmental stages and functioning levels interact with sensory profiles, offering insights to better understand the link between sensory processing anomalies and developmental trajectories in children and adolescents with ASD.

## 2 Materials and methods

### 2.1 Study sample

This is a monocentric retrospective observational study. Subject recruitment was conducted at the Child Neuropsychiatry Unit of the Federico II University of Naples. The inclusion criteria were defined as: comprehensive clinical evaluation resulted in an established diagnosis of ASD according to DSM-5 criteria, including the administration of a semi-structured ADOS-2 observation, which yielded a positive result; signed informed consent from parents/caregivers. Among individuals younger than 30 months, those who were classified as “at risk” on the semi-structured ADOS-2 Toddler Module were included, provided that their diagnosis was subsequently confirmed through a comprehensive clinical evaluation repeated after 31 months of age. The following exclusion criteria were also applied: presence of sensory organ pathologies (e.g., blindness, hearing loss); presence of documented genetic disorder; presence of documented neurological disorder (e.g., epilepsy, cerebral palsy); ongoing psychopharmacological treatment; withdrawal of previously provided informed consent. Eligible subjects were selected within a timeframe between January 2021 and December 2024. For all enrolled subjects, demographic data (age in months at the moment of the evaluation, sex), diagnosis and psychometric characteristics were collected.

### 2.2 Clinical and psychometric characterization

In order to pursue the aims of the study, information related to the clinical and psychometric characterization of the included subjects was retrieved. Specifically, the following information was collected:

Adaptive Profile: this was evaluated using the Vineland Adaptive Behaviors Scale - Second Edition (VABS-II), Italian version. This tool consists of a semi-structured interview administered to the subjects’ parents/caregivers. The VABS-II explores personal and social autonomies across four distinct domains (Communication, Daily Living Skills, Socialization, Motor Skills). The Adaptive Quotient, which reflects the overall level of autonomy of the individual subjects, was derived from this assessment and used as a predictor in the analysis. This score has a distribution comparable to a standardized normal distribution, with a mean of 100 and a standard deviation of 15. (12).
Developmental and Cognitive Profile: data regarding developmental profiles (for younger subjects) or cognitive profiles (for older, compliant subjects) were obtained for all participants. The Griffiths Scales of Child Development, 3rd Edition (Griffiths-III) was administered to assess the developmental profile. This tool evaluates child skills across five specific domains: Foundations of Learning, Language and Communication, Eye and Hand Coordination, Personal-Social-Emotional, and Gross Motor. These five domains are then combined into an overall developmental score. (13). Cognitive functioning was assessed using the Leiter International Performance Scale, Third Edition (Leiter-3) for non-verbal assessment (used for children with co-occurring language disorders or limited cooperation) (14) and the Wechsler Intelligence Scale for Children-fourth edition (WISC-IV) for verbal children capable of complying with the testing setting (15). The resulting general scores, the Griffiths General Development Quotient and Leiter-III or WISC-IV Intelligence Quotient were used as a predictors in the analysis. All three indicators are distributed according to a standardized normal distribution. Although the measurement of the developmental quotient is not entirely equivalent to that of an intelligence quotient, the developmental quotient represents the most appropriate measure of overall cognitive functioning in cases where an intelligence test cannot be administered, such as in preschool-aged patients. Studies in the literature have demonstrated both a high level of consistency between developmental and intellectual measures assessed concurrently (16), and that developmental measures are predictive of intellectual measures obtained at a later time (17).
Autistic Symptomatology: The severity of autistic symptoms was assessed using the Autism Diagnostic Observation Schedule – Second Edition (ADOS-2). This instrument evaluates ASD symptomatology across both the social-relational domain and the area of restricted and repetitive interests/behaviors (18). The ADOS-2 Total Score was derived/calculated for use in the final analysis. Modules were selected according to standardized procedures, based on the participant’s age and expressive language level, and symptom severity was summarized via the Calibrated Severity Score (19, 20).
Sensory Profile: Sensory processing patterns were assessed using the Sensory Profile 2 (SP-2). This caregiver questionnaire evaluates sensory processing variations in individuals with ASD via parental report, addressing both the involved sensory systems and the associated behavioral responses. The Sensory Profile 2 is a standardized assessment battery consisting of several questionnaires, differentiated according to the age range of administration and the intended respondents (caregivers and teachers). Specifically, it includes: Sensory Profile 2 Infant, administered to caregivers of infants from birth to 6 months; Sensory Profile 2 Toddler, administered to caregivers of children aged 7 to 35 months; Sensory Profile 2 Child, administered to caregivers of children and adolescents aged 3 years to 14 years and 11 months. Scores were derived for all domains provided by the instrument. Scores from scales that are not common to all forms for the age ranges considered were not included in the analysis. Based on the obtained scores, the results were used as ordinal measures classified according to the following interpretive categories: “Much Less Than Others” (below -2 SD); “Less Than Others” (between -2 SD and -1 SD); “Just Like the Majority of Others” (between -1 SD and +1 SD); “More Than Others” (between +1 SD and +2 SD); “Much More Than Others” (above +2 SD).” (21)

### 2.3 Statistical analysis

Firstly, descriptive analysis was conducted on the results describing the sensory profiles, as well as all proposed predictor variables.

Data cleaning procedures were applied to ensure numeric consistency of continuous variables, with non-numeric entries converted or treated as missing values. Missing data were handled using multiple imputation by chained equations (MICE), generating 5 completed datasets to account for uncertainty in the imputation process. Predictive mean matching (PMM) was used as the imputation method for all variables.

Ordinal outcomes were modeled using Bayesian cumulative logit models, which assume a latent continuous variable underlying the observed ordinal responses and estimate cumulative log-odds of response at each threshold. Each outcome was modeled as a function of sex, ADOS-2 Calibrated Severity Score (CSS), VABS-II Adaptive Score and Cognitive Quotient (development quotient or intelligence quotient), with age in months included as a continuous predictor via penalized splines (k = 5) to capture potential non-linear effects. Models were fitted across all 5 imputed datasets using the *brm_multiple* function in the *brms* package, which automatically pools posterior estimates across imputations.

Prior distributions were specified as weakly informative: normal priors (mean = 0, SD = 1) were placed on regression coefficients and normal priors (mean = 0, SD = 2) on intercept parameters. Four chains were run for 4000 iterations each. To ensure numerical stability, *adapt_delta* was set to 0.99 to reduce divergent transitions during sampling. Model convergence was assessed using R-hat statistics (all < 1.01) and effective sample sizes (> 1000). Posterior predictive checks indicated good agreement between observed and simulated data.

The non-linear effect of age on each outcome was examined through a series of spline-based analyses. First, the smooth term for age was visualized using conditional effects plots, with all other predictors held at their observed values. Second, the presence of a meaningful smooth effect was formally evaluated using a one-sided hypothesis test on the standard deviation of the spline coefficients, implemented via the *hypothesis* function in *brms*; the posterior distribution of this parameter was additionally summarized using its median and 95% credible interval (CI). Third, to characterize the shape of the developmental trajectory, posterior expected values were computed across a grid of 200 age values ranging from 16 to 179 months, with all covariates fixed at their median values (continuous variables) or modal value (sex). For each posterior draw, the expected score was calculated as the probability-weighted sum across response categories, yielding a continuous representation of the outcome as a function of age. From these trajectories, two summary statistics were derived: the age at which the expected score reached its maximum (peak age) and the overall range of the curve (difference between maximum and minimum expected score), both summarized as median and 95% CI across posterior draws.

Results are reported as median posterior estimates with 95% CI. Coefficients were exponentiated to obtain odds ratios (OR) with corresponding CI. Effects were considered statistically meaningful when the 95% CI excluded zero on the log-odds scale (i.e., the OR interval did not include 1). The non-linear effect of age was visualized using conditional effects plots.

No correction for multiple comparisons was applied. Within the Bayesian framework, posterior inference is not subject to the inflation of Type I error that motivates frequentist corrections such as Bonferroni adjustment, as CI directly quantify uncertainty about parameters without reference to a null hypothesis testing procedure. Furthermore, each outcome represents a conceptually distinct sensory processing domain and was modeled independently, such that the analyses do not constitute a family of related hypotheses for which multiplicity control would be warranted.

Descriptive statistics were conducted using Jamovi 2.6.2, while the analysis and the plotting of the graphs were conducted in R version 4.5.2 using the *brms* package with Stan as the backend sampler.

### 2.4 Ethical considerations

The study was conducted in accordance with the Declaration of Helsinki (22). The study was conducted according to principles of the Helsinki Declaration; ethical approval was obtained by the Ethics Committee **“**Campania 3**”** (research protocol No. 78/2024). A favorable opinion was obtained from an independent ethics committee before the study was carried out. For all subjects, informed consent was signed by the parents/caregivers before the start of the diagnostic assessments at our facility. This informed consent also provided availability for the anonymous use of the subjects**’** data for research purposes.

## 3 Results

### 3.1 Descriptive analysis

Among patients referred to the clinical unit for psychodiagnostic assessments, 159 subjects who met predefined inclusion and exclusion criteria were enrolled. These individuals had a median age of 47 months (IQR = 46), spanning a broad developmental range (16–179 months). The sex distribution was notably unbalanced, with a predominance of males (73%), which is consistent with the higher prevalence of neurodevelopmental conditions typically observed in males.

Adaptive functioning, as assessed by the VABS-II global score, had a median score of 70, indicating overall reduced adaptive abilities. Similarly, cognitive functioning measured using either developmental quotient or intelligence quotient had a median of 70 with an interquartile range of 46.5, suggesting substantial heterogeneity across participants. Cognitive assessments were obtained using multiple instruments (Griffiths III, Leiter III and WISC-IV), reflecting variability in age and ability levels within the sample.

Autism symptom severity, measured by ADOS-2 CSS, had a median of 7 (IQR = 3), indicating a generally moderate level of symptom severity, with scores ranging from 3 to 10.

Overall, the sample is characterized by considerable heterogeneity in age, cognitive functioning, adaptive skills and autism severity, with high data completeness across measures (≥96%), supporting the robustness of subsequent analyses.

Continuous variables were summarized as median and interquartile range; categorical variables were described using frequencies and percentages (**Table 1**).

**Table 1:** Characteristics of the sample.

Variable	Value
Sociodemographic	
Age in months, median (IQR)	47 (46)
Age range (months)	16-179
Sex, n (%)	
— Female	43 (27%)
— Male	116 (73%)
Clinical	
VABS-II Adaptive Score, median (IQR)	70 (30)
— n available (%)	153 (96.23%)
— range	20-123
Development Quotient/IQ, median (IQR)	70 (46.5)
— n available	156 (98.11%)
— n Griffiths III (% of total)	81 (50.94%)
— n Leiter III (% of total)	34 (21.38%)
— n WISC-IV (% of total)	41 (25.78%)
— range	20-125
ADOS-2 CSS, median (IQR)	7 (3)
— n available	154 (96.85%)
— range	3-10

Given the different age ranges considered, data from the Sensory Profile 2 Toddler and Child forms were used. The outcomes included in the analysis were those common to both forms, comprising sensory patterns (“seeking/active”, “avoiding/avoidant”, “sensitivity/sensory” and “registration/bystander”) and sensory processing systems (“auditory”, “visual”, “tactile”, “movement” and “oral”). **Table 2** reports the distribution of scores across the nine Sensory Profile 2 outcomes analyzed. Across all outcomes, the median score was “Just Like the Majority of Others”, with the exception of Movement Processing (median = “More Than Others”). Score distributions were right-skewed for most outcomes, with the majority of participants scoring at category “Just Like the Majority of Others”. Visual Processing showed the most concentrated distribution, with nearly 60% of participants scoring at category “Just Like the Majority of Others” and only 5% at the highest category. Movement Processing and Sensory Sensitivity showed the highest proportion of scores at category “Much More Than Others” (28.9% and 27.0%, respectively). A single value for the Sensory Seeking scale was missing (0.63%). Data for all other outcomes were complete and available for all 159 recruited participants.

**Table 2:** Absolute frequency of outcomes (% of total).

Outcome	Much less than others (below -2 SD)	Less than others (between -2 SD and -1 SD)	Just like the majority of others (between -1 SD and +1 SD)	More than others (between +1 SD and +2 SD)	Much more than others (above +2 SD)
Sensory Patterns					
Seeking/Active*	1 (0.6%)	7 (4.4%)	77 (48.7%)	43 (27.2%)	30 (19.0%)
Avoiding/Avoidant	3 (1.9%)	13 (8.2%)	85 (53.5%)	29 (18.2%)	29 (18.2%)
Sensivity/Sensory	0 (0.0%)	16 (10.1%)	77 (48.4%)	23 (14.5%)	43 (27.0%)
Registration/Bystander	7 (4.4%)	10 (6.3%)	75 (47.2%)	35 (22.0%)	32 (20.1%)
Sensory Systems					
Auditory Processing	2 (1.3%)	9 (5.7%)	73 (45.9%)	43 (27.0%)	32 (20.1%)
Visual Processing	2 (1.3%)	24 (15.1%)	95 (59.7%)	30 (18.9%)	8 (5.0%)
Tactile Processing	5 (3.1%)	12 (7.5%)	87 (54.7%)	26 (16.4%)	29 (18.2%)
Movement Processing	1 (0.6%)	7 (4.4%)	67 (42.1%)	38 (23.9%)	46 (28.9%)
Oral Processing	4 (2.5%)	7 (4.4%)	91 (57.2%)	21 (13.2%)	36 (22.6%)

*N = 158 for sensory seeking due to one missing observation.

### 3.2 Sensory patterns and sensory processing

**Figure 1:**
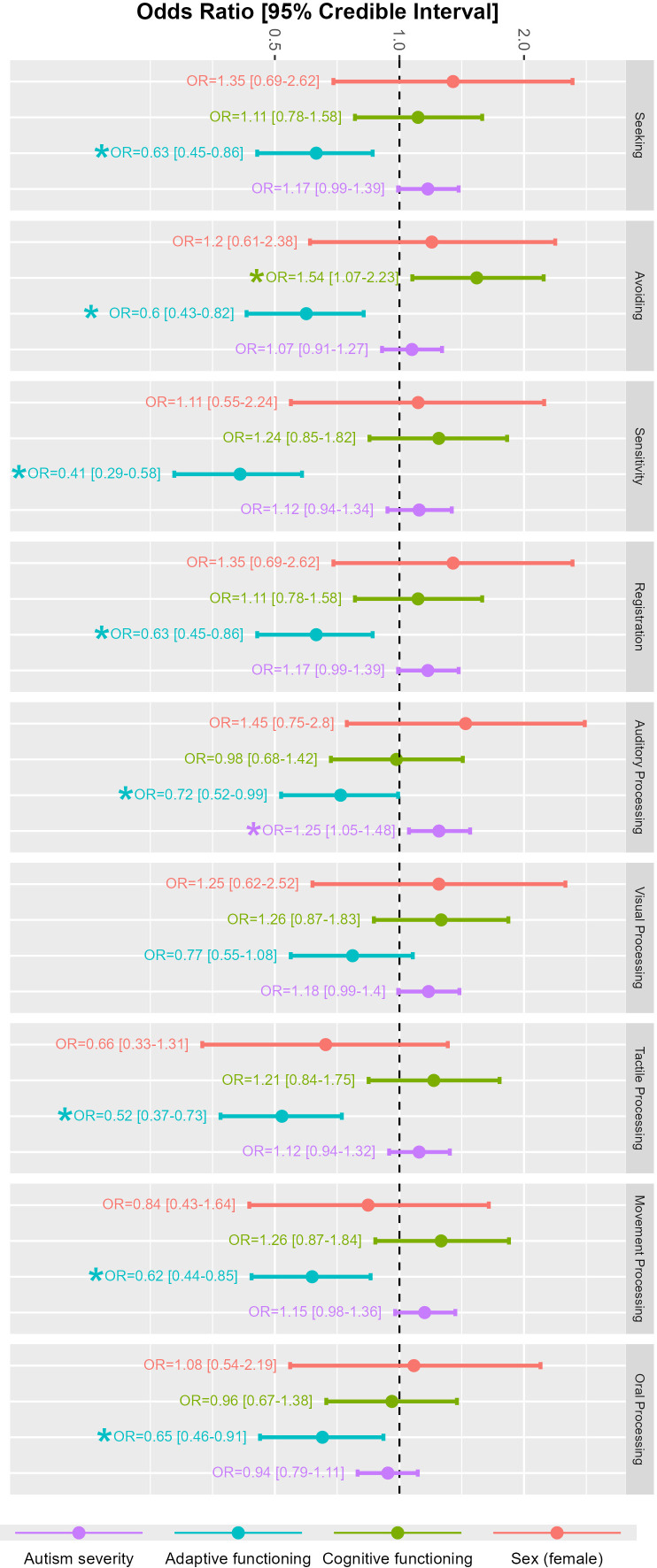
Association between predictors and sensory outcomes measured by Odds Ratio [95% CI]. Effects were considered statistically meaningful when the OR 95% CI did not include 1. Statistically significant effects are marked with an asterisk (*). The dashed line represents an Odds Ratio of 1, indicating no effect.

**Figure 1** reports the odds ratios and 95% CIs for all predictors across the nine sensory outcomes.

Overall, the Vineland Adaptive Behavior Scales (VABS) total score emerged as the most consistent predictor across models, with higher adaptive functioning associated with lower odds of atypical sensory processing patterns, including sensory seeking, avoiding, sensitivity and low registration. The strongest association was observed for sensory sensitivity.

In contrast, autism severity (ADOS-II calibrated severity scores) and sex were not significantly associated with most outcomes. Development or Intelligence quotients were generally not related to sensory patterns, with the exception of sensory avoiding, for which higher cognitive functioning was associated with increased odds of elevated scores.

Across specific sensory domains, adaptive functioning remained negatively associated with most outcomes, including auditory, tactile, movement, and oral processing. No predictors showed meaningful associations with visual processing. The only additional significant finding was observed for auditory processing, where higher autism severity was associated with increased odds of atypical scores.

### 3.3 Non-linear trajectories of sensory processing across age

To examine the relationship between age at evaluation and the nine Sensory Profile 2 outcomes, we modeled age (in months) as a smooth spline term within each Bayesian cumulative logistic regression model. The non-linearity of each trajectory was assessed by examining the posterior distribution of the smooth term standard deviation (*sds*), which quantifies the amount of variance captured by the spline. A CI for *sds* entirely above zero indicates that the non-linear component contributes meaningfully to the model.

Across all nine domains, the posterior probability that *sds* > 0 was 1.00, and the 95% CI excluded zero, supporting the presence of non-linear age-related trajectories in all sensory processing domains. However, the magnitude and precision of these effects varied substantially across domains.

The strongest and most precisely estimated non-linear effects were observed for Sensory Seeking (*sds*: median = 2.64, 95% CI [0.24-7.86]; curve variation: median = 0.61, 95% CI [0.45-0.73]) and Sensory Sensitivity (sds: median = 1.56, 95% CI [0.08-5.85]; curve variation: median = 0.60, 95% CI [0.46-0.73]). Visual Processing also showed a robust effect with the most precisely estimated variation across all domains (median = 0.61, 95% CI [0.52-0.69]).

Trajectory shapes differed markedly across domains. Sensory Seeking and Auditory Processing exhibited early peak trajectories, with posterior median peak ages of 61 months (95% CI [16-127]) and 63 months (95% CI [16-179]) respectively, suggesting that scores in these domains reach their maximum during the preschool period before declining. In contrast, Sensory Sensitivity, Sensory Avoidance, Visual Processing and Oral Processing showed later peak trajectories, with posterior median peak ages ranging from 104 to 146 months, indicating a more protracted developmental course extending into middle childhood and early adolescence. Sensory Registration showed an intermediate peak at 89 months (95% CI [16-179]).

Notably, Movement Processing displayed a qualitatively distinct pattern from all other domains, characterized by a monotonically decreasing trajectory across the full age range, with the posterior median peak collapsing to the lower boundary of the observed range (16 months). This suggests that vestibular and proprioceptive processing scores, as measured by the Sensory Profile 2, are highest in the youngest children and decline progressively with age.

It should be noted that for three domains — Tactile Processing, Sensory Avoidance and Oral Processing — the CIs for curve variation included values close to zero (lower bounds: 0.042, 0.036 and 0.044 respectively) and *sds* estimates were among the lowest observed (medians: 1.16, 0.93 and 1.04).

**Figure 2:**
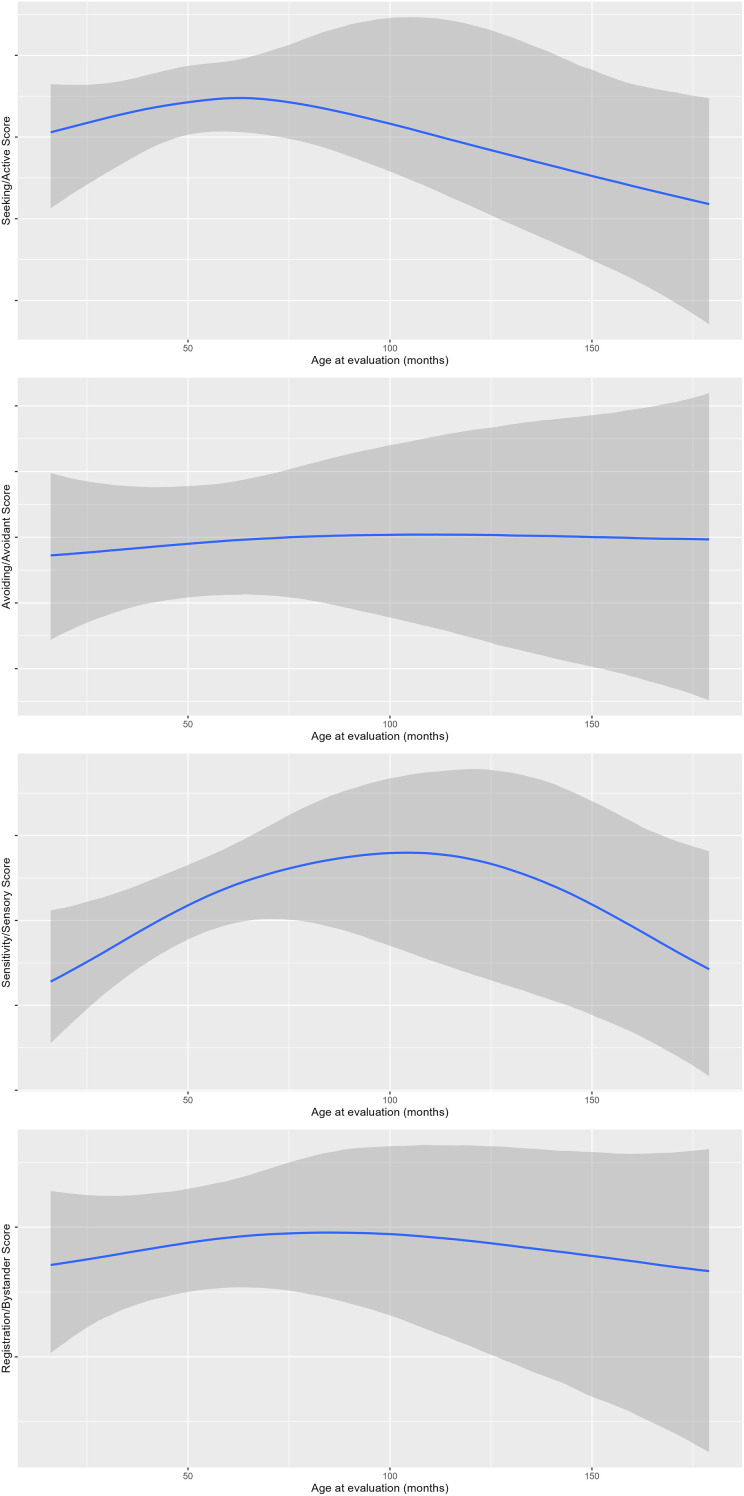
Conditional effects of age in months on patterns when other covariates (Sex, Cognitive functioning, Adaptive functioning and Autism severity) were held at their median or modal values: Sensory Seeking Sensory Avoiding, Sensory Sensitivity and Sensory Registration. Posterior mean spline trajectories (blue lines) with 95% credible intervals (shaded) for each SP2 domain. Non-linear age effects were credible across all patterns (P[sds > 0] = 1.00).

**Figure 3:**
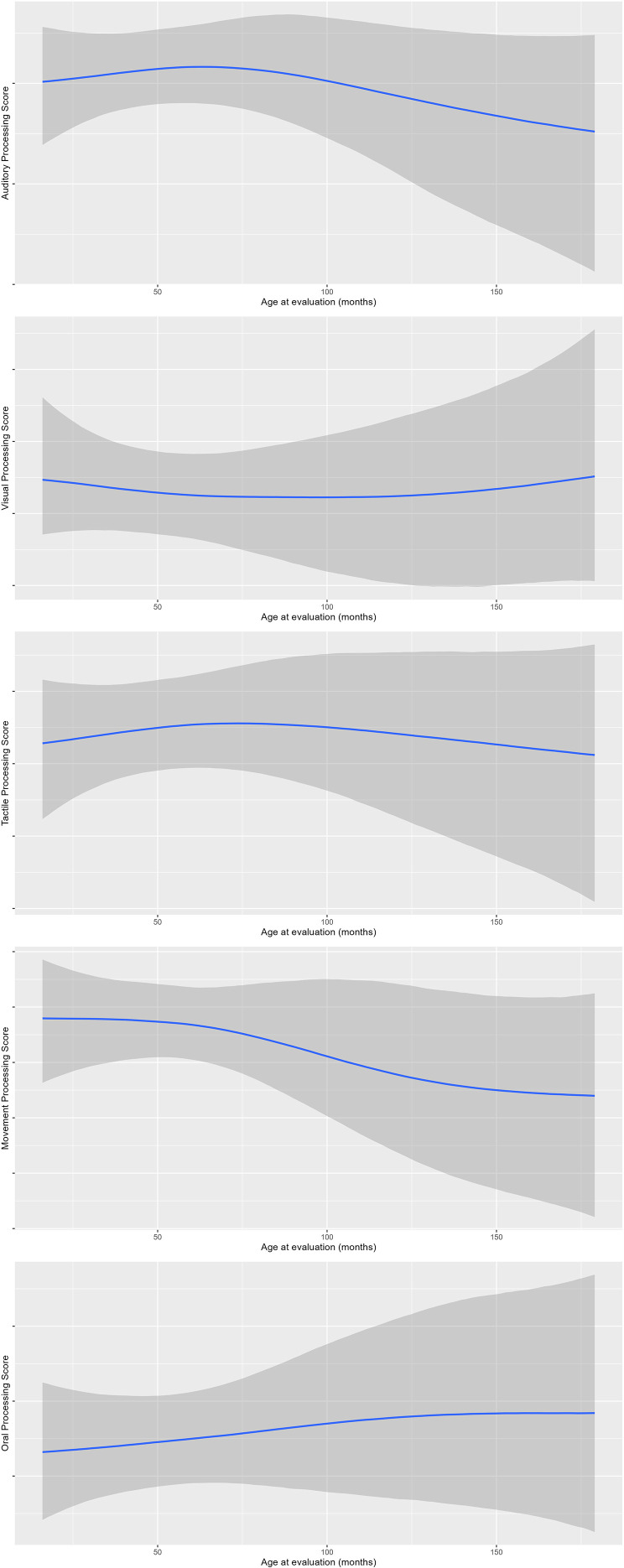
Conditional effects of age in months on sensory systems when other covariates (sex, cognitive functioning, adaptive functioning and autism severity) were held at their median or modal values: auditory processing, visual processing, tactile processing, movement processing and oral processing.

**Figures 2** and **3** display the conditional effects of age in months on each of the nine outcomes, arranged in two parts: sensory patterns and sensory processing patterns. All covariates were held at their median or modal values. Table 3 reports the peak age, curve range and spline standard deviation for each outcome with corresponding 95% CIs.

## 4 Discussion and conclusions

The results of the present study highlight how the sensory profile and behavioral patterns associated of ASD children vary as a function of age, adaptive functioning, cognitive functioning and autism symptomatology, confirming the complexity and heterogeneity of clinical manifestations.

Overall, it was found that the adaptive profile is a strong predictor of atypical sensory processing. Specifically, better scores on the VABS-II tool were associated to lower outcome scores, with a reduced likelihood of falling into the “more than others” and “much more than others” score categories and consequently fewer sensory anomalies, regarding both the type of anomaly and the sensory channel involved (auditory, tactile, oral, kinesthetic).

The link between adaptive functioning and sensory profile, confirmed by the effect of the VABS-II as a predictor, may suggest a bidirectional interaction: on one hand, alterations in sensory processing can hinder the development of autonomy and social skills; on the other hand, a lower adaptive level can exacerbate the perception of sensory difficulties by caregivers. These findings are partially consistent with the existing literature, which reports similar results in ASD populations of different ages (23, 24), while in other cases the findings appear to be inconsistent. Specifically, the studies by Dellapiazza F. et al. and Nieto C. et al. failed to identify a direct and significant relationship between sensory processing and adaptive functioning, despite observing an impact on maladaptive behaviors (4, 25).

The diversity of the highlighted results can also be interpreted in light of the tendency to integrate maladaptive behaviors into the concept of the adaptive profile. As highlighted by Dellapiazza and colleagues, most studies have observed a worsening of maladaptive behaviors (also including self-injury behaviors) rather than an impact on daily living skills (26).

Only a few studies, instead, have focused on the domain of daily living skills, observing their worsening in the presence of marked sensory anomalies (27). Some studies have also highlighted a negative impact of sensory anomalies on the communicative skills of ASD subjects (28).

Furthermore, in our sample, no strong impact of the cognitive profile or autistic symptomatology on the sensory profile was found. These aspects appear overall in line with what is reported in the literature (24). Nevertheless, analyzing the results specifically, two interesting aspects emerge. The first relates to the presence of greater avoidance behaviors in subjects with a higher IQ. As also described by Dunn W., one of the fundamental mechanisms in determining sensory response modes is linked to personal self-regulation strategies (29).

Therefore, this result could suggest that a higher IQ might favor better self-regulation strategies, leading to more frequent avoidance behaviors rather than sensory hyper-reactivity or sensory seeking behaviors. This association has also been described in the literature (30).

Moreover, according to a recent article by Kadlaskar G. et al., avoiding strategies are more prevalent also in the non-ASD population and could represent a way to cope with stress and anxiety in sensory overwhelming situations (31).

The association found between autistic symptoms and sensory processing abilities is also of particular interest. This finding is consistent with existing literature, especially regarding the impact that sensory processing alterations can have on social symptoms (32).

The Bayesian analysis results reveal that age acts as a global modifier of the sensory profile. Specifically, it was found that sensory profiles do not follow a monotonic trend; instead, sensory anomalies tend to cluster around two age peaks. During the preschool years, Sensory Seeking behaviors and auditory processing difficulties are more prominently represented. Conversely, between the ages of 8 and 12, hypersensitivity and avoidance behaviors tend to prevail, with visual and oral sensory channels showing the greatest anomalies. From a neurobiological perspective, it has been observed that neural reactivity—particularly within the sensory cortex and the amygdala—decreases as age increases in ASD individuals, even though this trend is not reflected by changes in the severity of clinical symptoms (33). To date, results from longitudinal studies regarding the sensory profile remain mixed and inconsistent. Specifically, some studies have highlighted that hyper-reactivity symptoms tend to improve over time (34), while others have shown relative stability of these features over time (35, 36). Notably, McCormick et al. (35) demonstrated that intellectual level may serve as an additional modifier during preschool and school age. In light of this evidence, the findings of the present article highlight a non-monotonic trend in the sensory profile, partially diverging from initial expectations. This discrepancy may be attributed to the different study designs employed (cross-sectional vs. longitudinal). Therefore, while age may represent a global modifier of sensory processing over time, the design of the present study does not allow for an accurate estimation of the expected individual evolution based on other clinical factors. While the posterior probability of a non-flat trajectory remained high for these domains, the magnitude of the age-related effect is uncertain and should be interpreted with caution.

## Conclusion

The present study aimed to investigate the association and potential impact of several clinical variables on the sensory profile of individuals with ASD across different age spans, using a retrospective observational structure. The findings underscored the primary influence of adaptive functioning, as measured by the VABS-II, on sensory processing, while autism symptom severity and cognitive profiles exhibited a more limited impact. Sex did not emerge as a significant predictor. Furthermore, the Bayesian model identified a non-monotonic trajectory, with fluctuating peaks across distinct sensory modalities and associated behaviors depending on the participant’s age. The main limitations of the study are attributed to the retrospective nature of data collection, as well as the lack of longitudinal observation of these aspects. Therefore, further longitudinal studies are considered necessary to investigate the association between specific clinical variables and the sensory profile expressed by individuals with ASD over time.

## Data Availability

The original contributions presented in the study are included in the article/supplementary material. Further inquiries can be directed to the corresponding author.

## References

[1] American Psychiatric Association . DSM-5-TR: diagnostic and statistical manual of mental disorders, Fifth Edition. (2022), Text Revision.

[2] Sivapalan S Sivayokan B Raveenthiran K Sivayokan S . Sensory issues and their impact among autistic children: A cross-sectional study in Northern Sri Lanka. Cureus. (2024) 16:e72130. doi: 10.7759/cureus.72130. PMID: 39575000 PMC11580710

[3] Patil O Kaple M . Sensory processing differences in individuals with autism spectrum disorder: A narrative review of underlying mechanisms and sensory-based interventions. Cureus. (2023) 15:e48020. doi: 10.7759/cureus.48020. PMID: 38034138 PMC10687592

[4] Dellapiazza F Michelon C Oreve MJ Robel L Schoenberger M Chatel C . The impact of atypical sensory processing on adaptive functioning and maladaptive behaviors in autism spectrum disorder during childhood: results from the ELENA cohort. J Autism Dev Disord. (2020) 50:2142–52. doi: 10.1007/s10803-019-03970-w. PMID: 30868365

[5] Khaledi H Aghaz A Mohammadi A Dadgar H Meftahi G . The relationship between communicationskills, sensory difficulties, and anxiety in children with autism spectrum disorder. Middle East Curr Psychiatry. (2022) 29:69. doi: 10.1186/s43045-022-00236-7. PMID: 38164791

[6] Crasta JE Salzinger E Lin MH Gavin WJ Davies PL . Sensory processing and attention profiles among children with sensory processing disorders and autism spectrum disorders. Front Integr Neurosci. (2020). doi: 10.3389/fnint.2020.00022. PMID: 32431600 PMC7214749

[7] Dunn W . The sensations of everyday life: empirical, theoretical, and pragmatic considerations. Am J Occup therapy: Off Publ Am Occup Ther Assoc. (2001) 55:608–20. doi: 10.5014/ajot.55.6.608. PMID: 12959225

[8] Tomchek SD Little LM Dunn W . Sensory pattern contributions to developmental performance in children with autism spectrum disorder. Am J Occup therapy: Off Publ Am Occup Ther Assoc. (2015) 69:6905185040p1–6905185040p10. doi: 10.5014/ajot.2015.018044. PMID: 26356661

[9] Osório JMA Rodríguez-Herreros B Richetin S Junod V Romascano D Pittet V . Sex differences in sensory processing in children with autism spectrum disorder. Autism Res. (2021) 14:2412–23. doi: 10.1002/aur.2580. PMID: 34288517 PMC9290069

[10] Pastor-Cerezuela G Fernández-Andrés MI Sanz-Cervera P Marín-Suelves D . The impact of sensory processing on executive and cognitive functions in children with autism spectrum disorder in the school context. Res Dev Disabil. (2020) 96:103540. doi: 10.1016/j.ridd.2019.103540. PMID: 31862533

[11] van den Boogert F Sizoo B Spaan P Tolstra S Bouman YHA Hoogendijk WJG . Sensory processing and aggressive behavior in adults with autism spectrum disorder. Brain Sci. (2021) 11:95. doi: 10.3390/brainsci11010095. PMID: 33466570 PMC7828723

[12] Sparrow SS Cicchetti DV Balla DA . Vineland-II: vineland adaptive behavior scales. 2nd ed. Giunti Psychometrics (2016). Survey Forms.

[13] Stroud L Foxcroft C Green E Bloomfield S Cronje J Hurter K . Griffiths scales of child development. 3rd Edition. Hogrefe Ltd (2016). Manual.

[14] Roid GH Miller LJ Pomplun M Koch C . Leiter international performance scale. In: (Leiter-3), 3rd ed. Western Psychological Services (WPS (2013).

[15] Wechsler D . Wechsler intelligence scale for children. In: (WISC-IV; giunti psychometrics 2012), 4th ed. (2003).

[16] Portoghese C Buttiglione M De Giacomo A Lafortezza M Lecce PA Martinelli D . Leiter-R versus developmental quotient for estimating cognitive function in preschoolers with pervasive developmental disorders. Neuropsychiatr Dis Treat. (2010) 6:337–42. doi: 10.2147/ndt.s10657. PMID: 20856598 PMC2938283

[17] Sutcliffe AG Soo A Barnes J . Predictive value of developmental testing in the second year for cognitive development at five years of age. Pediatr Rep. (2010) 2:e15. doi: 10.4081/pr.2010.e15. PMID: 21589828 PMC3093999

[18] Lord C Luyster RJ Gotham KO Guthrie W . Autism diagnostic observation schedule. In: (ADOS-2), 2nd ed. (2012). doi: 10.1007/978-0-387-79948-3_1520

[19] Gotham K Pickles A Lord C . Standardizing ADOS scores for a measure of severity in autism spectrum disorders. J Autism Dev Disord. (2009) 39:693–705. doi: 10.1007/s10803-008-0674-3. PMID: 19082876 PMC2922918

[20] Esler AN Bal VH Guthrie W Wetherby A Ellis Weismer S Lord C . The autism diagnostic observation schedule, toddler module: standardized severity scores. J Autism Dev Disord. (2015) 45:2704–20. doi: 10.1007/s10803-015-2432-7. PMID: 25832801 PMC4898775

[21] Dunn W . Sensory profile 2. Adattamento italiano di Ilaria Basadonne: Adattamento italiano di Ilaria Basadonne (2020). doi: 10.1037/t15155-000

[22] World Medical Association . World Medical Association Declaration of Helsinki: ethical principles for medical research involving human subjects. JAMA. (2013) 310(20):2191–4. doi: 10.1001/jama.2013.281053. PMID: 24141714

[23] Williams KL Kirby AV Watson LR Sideris J Bulluck J Baranek GT . Sensory features as predictors of adaptive behaviors: A comparative longitudinal study of children with autism spectrum disorder and other developmental disabilities. Res Dev Disabil. (2018) 81:103–12. doi: 10.1016/j.ridd.2018.07.002. PMID: 30060977 PMC7473611

[24] Basadonne I Passani V Cagiano R Nencioli R Costanzo V Giorgetti F . Sensory processing correlates with adaptive behaviors but not with symptom severity in Italian children with autism spectrum disorders. Bollettino di Psicologia Applicata. (2025). doi: 10.26387/bpa.2025.00001

[25] Nieto C López B Gandía H . Relationships between atypical sensory processing patterns, maladaptive behaviour and maternal stress in Spanish children with autism spectrum disorder. J Intellectual Disability Res. (2017) 61:1140–50. doi: 10.1111/jir.12435. PMID: 29154486

[26] Dellapiazza F Vernhet C Blanc N Miot S Schmidt R Baghdadli A . Links between sensory processing, adaptive behaviours, and attention in children with autism spectrum disorder: A systematic review. Psychiatry Res. (2018) 270:78–88. doi: 10.1016/j.psychres.2018.09.023. PMID: 30245380

[27] Jasmin E Couture M McKinley P Reid G Fombonne E Gisel E . Sensori-motor and daily living skills of preschool children with autism spectrum disorders. J Autism Dev Disord. (2009) 39:231–41. doi: 10.1007/s10803-008-0617-z. PMID: 18629623

[28] Lane AE Young RL Baker AE Angley MT . Sensory processing subtypes in autism: association with adaptive behavior. J Autism Dev Disord. (2010) 40:112–22. doi: 10.1007/s10803-009-0840-2. PMID: 19644746

[29] Dunn W . Supporting children to participate successfully in everyday life by using sensory processing knowledge. Infants Young Children. (2007) 20:84–101. doi: 10.1097/01.IYC.0000264477.05076.5d. PMID: 33079766

[30] Shulman C Peretz-Bornstein Y Kagya S Ben Shalom D . Sensory subgroups in autism: The role of cognitive abilities. J Autism Dev Disord. (2026). doi: 10.1007/s10803-026-07230-6. PMID: 41609989

[31] Kadlaskar G King SE Stewart JR . Sensory reactivity in children referred for autism evaluation: Associations with autism symptoms and adaptive skills. Brain Sci. (2026) 16:310. doi: 10.3390/brainsci16030310. PMID: 41892653 PMC13025295

[32] Poulsen R Williams Z Dwyer P Pellicano E Sowman PF McAlpine D . How auditory processing influences the autistic profile: A review. Autism Res. (2024) 17:2452–70. doi: 10.1002/aur.3259. PMID: 39552096 PMC11638897

[33] Cakar ME Cummings KK Bookheimer SY Dapretto M Green SA . Age-related changes in neural responses to sensory stimulation in autism: a cross-sectional study. Mol Autism. (2023) 14:38. doi: 10.1186/s13229-023-00571-4. PMID: 37817282 PMC10566124

[34] Dwyer P Saron CD Rivera SM . Identification of longitudinal sensory subtypes in typical development and autism spectrum development using growth mixture modelling. Res Autism Spectr Disord. (2020) 78:101645. doi: 10.1016/j.rasd.2020.101645. PMID: 32944065 PMC7491753

[35] McCormick C Hepburn S Young GS Rogers SJ . Sensory symptoms in children with autism spectrum disorder, other developmental disorders and typical development: A longitudinal study. Autism: Int J Res Pract. (2016) 20:572–9. doi: 10.1177/1362361315599755 PMC4918912 26395236

[36] Baranek GT Carlson M Sideris J Kirby AV Watson LR Williams KL . Longitudinal assessment of stability of sensory features in children with autism spectrum disorder or other developmental disabilities. Autism Res. (2019) 12:100–11. doi: 10.1002/aur.2008. PMID: 30194913 PMC6500492

